# Targeted-release budesonide: A comprehensive review on its potential in IgA nephropathy

**DOI:** 10.1016/j.heliyon.2025.e42729

**Published:** 2025-02-15

**Authors:** Fei-fan Qi, Hui-qin Zeng, Jian-jiang Zhang

**Affiliations:** Department of Pediatrics, The First Affiliated Hospital of Zhengzhou University, Clinical Center of Pediatric Nephrology of Henan Province, Zhengzhou, 450052, China

**Keywords:** Targeted-release budesonide, IgA nephropathy, Proteinuria, Review

## Abstract

IgA nephropathy (IgAN) is characterized by the presence of IgA deposits in the glomerular mesangium, representing a prevalent form of primary glomerulonephritis worldwide. This condition is associated with a significant risk of progression to end-stage renal disease (ESRD). Hypertension, proteinuria, and reduced glomerular filtration rate (GFR) are established risk factors. Although angiotensin-converting enzyme inhibitors (ACEIs) and angiotensin receptor blockers (ARBs) are currently the first-line treatments, they do not adequately mitigate the risk of disease progression. Budesonide is a potent corticosteroid that has been utilized for many years in the treatment of inflammatory diseases. Recently, a novel formulation, targeted-release budesonide (TRF-budesonide), was developed to facilitate drug release specifically in the distal ileum to treat IgAN. This review synthesizes the existing evidence on the impact of TRF-budesonide in IgAN, covering its pathogenesis, efficacy, and safety. It also explores comparisons between TRF-budesonide and other therapeutic options, highlighting the advantages of TRF-budesonide in reducing proteinuria and preserving renal function. While TRF-budesonide has demonstrated promising efficacy and safety in short- and medium-term studies, showcasing its potential as a valuable treatment option for IgAN, further high-quality randomized controlled trials are needed to comprehensively evaluate its long-term efficacy and safety. Such research will pave the way for more personalized and precise treatment options for patients with IgAN.

## Highlights

• Our article suggested targeted release budesonide (TRF-budesonide) as a potential first-line treatment for mild to moderate IgA nephropathy (IgAN), minimizing systemic side effects.
• Our comprehensive review of clinical trials and studies supported the efficacy and safety of TRF-budesonide in reducing proteinuria and stabilizing renal function in IgAN patients.
• We compared TRF-budesonide with systemic corticosteroids and other alternative treatments, highlighting its potential as a safer and more targeted therapeutic option.
• Our review covered IgAN pathogenesis, the mechanism of action of trf-budesonide, clinical trials and studies, comparisons with systemic corticosteroids and other alternative treatments, and long-term safety and efficacy.

## 1 Introduction

Primary IgA nephropathy (IgAN) is a condition characterized by the deposition of IgA in the glomerular mesangium. This condition can lead to episodic gross hematuria, persistent microscopic hematuria, and varying degrees of proteinuria. Additionally, some patients may develop nephrotic syndrome, acute or rapidly progressive nephritic syndrome, hypertension, and decreased renal function. First described by Jean Berger in 1968, this condition has since been referred to as "Berger's disease” [1]. IgAN is recognized as a common form of primary glomerulonephritis worldwide, with an annual incidence rate ranging from 1 to 2.5 per 100,000 individuals [2,3]. Up to 40 % of patients may develop end-stage renal disease (ESRD) within 20–25 years, necessitating renal replacement therapy [4,5]. Ethnicity influences the risk of disease progression, with East Asians being more susceptible than individuals of African descent [6]. Asian patients exhibit a higher risk of acute lesions and poorer renal outcomes compared to Caucasians [7,8]. Recognized risk factors for disease progression include hypertension, persistent proteinuria exceeding 1 g/day, smoking and reduced glomerular filtration rate (GFR). Notably, persistent microscopic hematuria is now acknowledged as a marker of disease activity [[9], [10], [11]].

In the current medical landscape, angiotensin-converting enzyme inhibitors (ACEIs) and angiotensin receptor blockers (ARBs) are established as the primary treatments for IgAN, as indicated in Ref. [3]. The globally recognized Kidney Disease Improving Global Outcomes (KDIGO) guidelines recommend that all patients with proteinuria exceeding 0.5 g/day receive treatment with ACEIs or ARBs, regardless of their blood pressure levels, starting at the time of diagnosis and continuing throughout follow-up. For high-risk patients with proteinuria greater than 1 g/day, despite optimized supportive care, a 6-month course of glucocorticoid therapy may be considered [12]. However, high-dose systemic corticosteroids increase the risk of adverse events, including infections, hypertension, weight gain, diabetes, and osteoporosis [[13], [14], [15]]. The Supportive Versus Immunosuppressive Therapy for the Treatment of Progressive IgA Nephropathy (STOP-IgAN) and the Therapeutic Evaluation of Steroids in IgA Nephropathy Global (TESTING) trials [16,17] confirmed that immunosuppressants and systemic corticosteroids can effectively reduce proteinuria in patients with IgAN. However, these treatments are also associated with a high incidence of adverse events, including opportunistic infections, hematologic toxicity, and metabolic disturbances, leading to uncertainty and debate within the medical community regarding the optimal treatment strategy for IgAN. Recently, a new oral targeted-release glucocorticoid formulation, TRF-budesonide, was developed to release the drug in the distal ileum. Richard Lafayette proposed that budesonide can target the distal ileum, which is rich in Peyer's patches, to protect renal function in adults with primary IgAN who are at risk of rapid progression [18].

This review examines the impact of TRF-budesonide on IgAN up to July 2024, assessing the pathogenesis of IgAN and the efficacy and safety of TRF-budesonide in treating IgAN.

## 2 The pathogenesis of IgAN

**Fig. 1:**
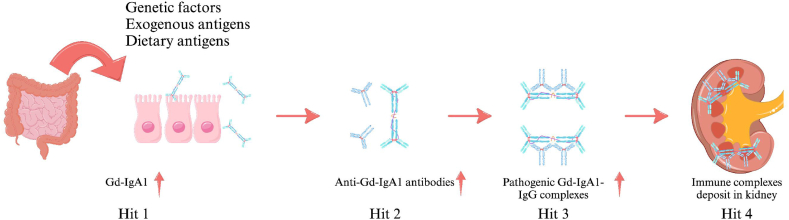
The four-hit hypothesis of IgAN.

IgAN is characterized by the mesangial deposition of immune complexes that contain IgA1 and IgA1-bound IgG antibodies. IgA, primarily located on mucosal surfaces, plays a crucial role in protecting against antigens [3,19]. The poor O-galactosylation of IgA leads to the formation of galactose-deficient IgA1 (Gd-IgA1)-IgG immune complexes. These complexes contribute to kidney damage [19,20]. The four-hit hypothesis elucidates the formation of IgAN: (1) elevated circulating levels of Gd-IgA1 [19]; (2) the triggering of an autoimmune reaction and the production of anti-Gd-IgA1 antibodies [3]; (3) the formation of pathogenic Gd-IgA1-IgG complexes [3]; (4) the deposition of these complexes in the kidney, which activates the complement pathway and results in glomerular injury (Fig. 1).

Recent studies employing genome-wide association, proteomic analyses, and experimental models have elucidated the mechanisms underlying IgAN. Genetic susceptibility, mucosal immune responses, inflammatory pathways, complement activation, and both innate and adaptive immunity collectively contribute to the pathogenesis of IgAN [[21], [22], [23]]. The mucosal immune system of the gut, along with mucosal-derived Gd-IgA1, is considered critical to the primary pathogenesis of IgAN. Gd-IgA1, a specific immunoglobulin produced primarily by plasma cells located in various regions of the body, including the gut-associated lymphoid tissue (GALT), bone marrow, and lymph nodes, has been shown to exhibit a distinct deficiency in galactose [24]. Dysbiosis of gut microbiota and dysregulated mucosal immune responses can impair gut barrier function and immune responses, contributing to the development of IgAN [25]. Therefore, the gut-kidney axis is crucial in the pathogenesis of IgAN. Studies have explored the relationship between the gut microbiome and IgAN, revealing that dysbiosis of gut microbiota is associated with the clinical features of the disease [26]. Furthermore, Gd-IgA1 can form immune complexes with circulating anti-glycan IgG antibodies [[27], [28], [29]]. These complexes can bind to mesangial cells in the kidney, promoting proteinuria and fibrotic remodeling, which ultimately leads to a loss of renal function [27,29]. This underscores the role of Gd-IgA1 in IgAN and suggests that targeting the gut-associated lymphatic system with therapeutic agents may effectively treat the disease by reducing Gd-IgA1 production [30]. There are several treatment options available for IgA nephropathy, with the potential approaches summarized in Table 1.

**Table 1:** Possible treatment options for IgA nephropathy.

Drugs	Clinical Application	Advantages	Disadvantages or Side Effects
Budesonide	Mild to moderate IgAN patients, particularly for high-risk patients who experience severe side effects from traditional therapies	Fewer side effects, mainly acts on gut-associated immune responses	Acne, hypertension, facial swelling, and weight gain
Systemic Corticosteroids	Used in moderate to severe IgAN patients	Reduce proteinuria and delay renal function deterioration	Systemic adverse reactions are significant and may lead to increased risks of hyperglycemia, hypertension, osteoporosis, and infections
ACEI/ARB	As a base medication, suitable for most IgAN patients	Protects renal function, reduces proteinuria, high safety in long-term use	May cause hyperkalemia, low blood pressure
Mycophenolate Mofetil (MMF)	Used for patients unresponsive or intolerant to glucocorticoids	Protects renal function, reduces proteinuria, milder side effects	May cause gastrointestinal discomfort and increased infection risk
Cyclophosphamide	Used in severe cases or when other drugs are ineffective	Maybe effective for severe or rapidly progressive IgAN	Bone marrow suppression, carcinogenic risk
Hydroxychloroquine (HCQ)	Maybe benefit for IgAN in some studies, but further research is needed	Fewer side effects, suitable for long-term use	May cause retinal damage, requiring regular ophthalmological examinations
Telitacicept	Potential future treatment option	Acts on multiple immune system pathways, may help with IgA deposition	As a new drug, long-term safety and efficacy require further research

## 3 The pharmacological properties of budesonide

Budesonide, a second-generation synthetic glucocorticoid, is a medication characterized by low systemic absorption. It exhibits potent local anti-inflammatory effects, enabling the reduction of inflammatory mediators and the suppression of inflammatory cell activity [31]. As a local corticosteroid, budesonide demonstrates high specificity for target tissues and is released at designated sites, specifically the distal ileum, utilizing TarGeted Release for Inflammatory conditions of the GI Tract (TARGIT™ technology) to safeguard the renal function of adult patients with primary IgAN who are at risk of rapid disease progression [18].

TARGIT™ technology is an innovative oral drug delivery system developed by West Pharmaceutical Services. This technology is specifically designed for targeted drug delivery to certain sites within the gastrointestinal (GI) tract, particularly for targeted release in the colon area. TARGIT™ technology utilizes a pH-sensitive coating applied to injection-molded starch capsules, allowing the capsules to release the drug in the specific pH environment of the small intestine. This ensures that the drug acts directly on the affected area rather than systemically. This targeted release strategy is especially valuable for treating diseases such as IgAN. Additionally, TARGIT™ technology helps to enhance the bioavailability of drugs by protecting them from degradation by gastric acid and digestive enzymes, ensuring the drug remains stable before reaching its site of action [32].

Following oral administration, its systemic bioavailability is limited due to the first-pass effect, resulting in relatively low circulating concentrations in the body. This limitation contributes to a reduction in systemic side effects [33]. Its half-life (3–4 h) is marginally longer than that of other corticosteroids (2–3 h), facilitating the rapid relief of inflammatory symptoms. Nevertheless, continuous dosing remains necessary to sustain therapeutic effects [34,35]. Budesonide is available in multiple formulations, including inhalers, oral capsules, and targeted-release preparations, to address diverse treatment needs.

## 4 Mechanism of budesonide in the treatment of IgA nephropathy

**Fig. 2:**
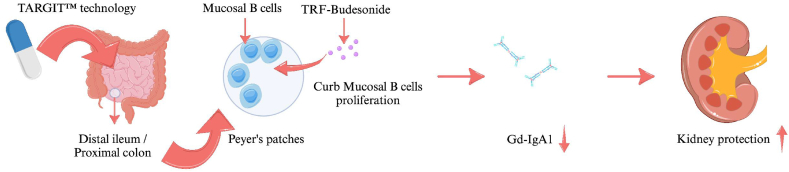
The mechanism of TRF-budesonide in IgAN.

As previously noted, IgA immunoglobulins are predominantly produced in mucosa-associated lymphoid tissue (MALT) [24], while Peyer's patches in the intestinal mucosa are components of gut-associated lymphoid tissue (GALT). These patches serve as antigen-sampling and induction sites and also contain mucosal B cells that express Gd-IgA1 [24]. TRF-budesonide facilitates the targeted delivery of medication to the distal ileum and proximal colon, where the density of Peyer's patches is greatest, utilizing the innovative TARGIT™ technology. This mechanism provides a local anti-inflammatory effect, effectively curbing the proliferation of mucosal B lymphocytes and Peyer's patches [32,36] (Fig. 2). Consequently, this reduction in GdgA1 production alleviates inflammation and immune complex deposition. In the gastrointestinal tract, approximately 70 % of TRF-budesonide releases its active compound in the distal ileum and proximal colon following absorption. The bioactive substance subsequently enters the systemic circulation, where it is initially metabolized by a group of enzymes collectively referred to as cytochrome P450. Prominent among these enzymes are CYP3A4 and CYP3A5; these enzymes convert the active compound into by-products with reduced glucocorticoid activity. Specifically, these enzymes catalyze the formation of 16α-hydroxyprednisone and 6β-hydroxybudesonide, both of which possess considerably less potency than the original compound. TRF-budesonide exhibits a bioavailability of 10 % in the systemic circulation. It demonstrates a stronger glucocorticoid effect than prednisone; however, it is safer for the long-term treatment of patients with IgAN due to its lower systemic bioavailability [32,36].

## 5 Clinical study and treatment results of budesonide

### 5.1 Literature search

To maximize the discovery of studies related to TRF-budesonide treatment for IgA nephropathy, we conducted a comprehensive review of three commonly used databases: PubMed, MEDLINE, and the Cochrane Library. The keywords included terms and synonyms of IgA nephropathy, Berger's Disease and Targeted Release Formulation of Budesonide. We incorporated clinical trials, observational studies, and case reports involving patients undergoing TRF-budesonide treatment for IgAN, culminating in a total of ten studies. This collection comprised four randomized controlled trials, one cohort study, one prospective study, and four case reports. Utilizing standardized data collection forms, we meticulously extracted the following information: the name of the first author, year of publication, study design, sample size and/or placebo group, administration details of TRF-budesonide, principal findings of each study, and any adverse events associated with TRF-budesonide. Given the variations in study design, interventions, and reported outcome measures, a meta-analysis was deemed unsuitable; hence, we concentrated on articulating the evidence-based studies and their findings within a comprehensive review. Table 2 summarizes the studies reporting TRF-Budesonide against IgAN.

**Table 2:** Characteristics and the main findings in the nine studies.

Authors	Study design	Study Group	Placebo Group	Daily Dosage	Main findings	Adverse events
Smerud et al. (2011) [37]	RCT	16 patients	NA	8 mg (Nefecon)	The median reduction in urine albumin (U-albumin) was 529 mg/day during the treatment period (P = 0.04), and after a follow-up of 2 months the median reduction in U-albumin a peaked at 40 %. The relative reduction in serum creatinine of 6 % from baseline after treatment (P = 0.003).	Abdominal pain (12.5 %)
Fellström et al. (2017) [38]	RCT	1st group: 48 patients; 2nd group: 51 patients	50 patients	1st group received 16 mg (Nefecon); 2nd group received 8 mg (Nefecon)	At 9 months, TRF-budesonide (16 mg/day plus 8 mg/day) was associated with a 24.4 % decrease from baseline in mean UPCR (p = 0.0066). At 9 months, the mean UPCR had decreased by 27.3 % in 48 patients who received 16 mg/day (p = 0.0092) and by 21.5 % in the 51 patients who received 8 mg/day (p = 0.0290); whereas the mean UPCR increased by 2.7 % in 50 patients who received the placebo.	Thirteen serious adverse events occurred, with two possibly related to TRF-budesonide: deep vein thrombosis (at a dose of 16 mg/day) and unexplained deterioration of renal function.
Ismail et al. (2020) [39]	Retrospective cohort	18 patients	18 patients (Systemic steroids group)	9 mg for 12 months, followed by 3 mg for another 12 months (Budenofalk)	The median reduction in proteinuria was 45 % in the TRF-budesonide group and 11 % in the corticosteroid group at 24 months (P = 0.009).	Nausea (6 %), Viral upper respiratory tract infection (6 %) Oral candidiasis (6 %)
Lafayette et al. (2023) [18]	RCT	182 patients	182 patients	16 mg (Nefecon)	Within two years, the time-weighted average of eGFR demonstrated a statistically significant benefit of Nefecon treatment compared to placebo (with a difference of 5.05 mL/min/1.73 m^2^ (p < 0.0001)).	Hypertension, peripheral oedema, muscle spasms, and acne were reversible.
Obrisca et al. (2023) [40]	a prospective study	32 patients	NA	9 mg/day for 12 month, subsequently tapered to 3 mg/day for another 12 months (Budenofalk)	Urinary protein excretion decreased from a baseline level of 1.89 ± 1.5 g/d to 0.5 ± 0.4 g/d after 36 months of treatment (p < 0.001). Budesonide therapy was associated with a significant reduction in proteinuria regardless of the baseline levels. Although there was a trend towards an increase in eGFR during the treatment period, the average eGFR returned to baseline levels during the follow-up at 12 months post-treatment.	NA
Zhang H et al. (2024) [41]	RCT	62 patients	30 patients	16 mg (Nefecon)	The time-weighted average eGFR over two years showed a significant advantage compared to the placebo group, with a mean difference of 9.6 mL/min/1.73 m^2^. The UPCR decreased by 31 % at 9 months and by 43 % at 24 months.	The incidence of serious adverse events was low, and no severe infections requiring hospitalization were reported.
Venettacci et al. (2018) [42]	Case report	a 12-year-old boy	NA	TRF-Budesonide 3 mg/day was given orally for 4 weeks, then increased to 6 mg daily.	His UPCR reduced to 69 mg/mmol and serum creatinine falled to 69 mmol/L, and no side effects were reported.	NA
Lingaraj et al. (2020) [43]	Case report	a 34-year-old male patient	NA	TRF-budesonide 9 mg/day was given orally for 9 months, then reduced to 3 mg/d and continued.	After 6 months of treatment, the patient's serum creatinine was 1.1 mg/dL and urine tests were normal, with no reported side effects during treatment.	NA
Sladowska-Kozłowska et al. (2022) [44]	Case report	a 13-year-old boy	NA	TRF-budesonide 15 mg/day was given orally for 6 months.	After 6 months, the patient's UPCR reduced below 100 g/mol and serum creatinine to 1.6 mg/dL. Budesonide was well tolerated, and no side effects were observed.	NA
Antonucci, L et al. (2023) [45]	Case report	a 13-year-old boy	NA	TRF-budesonide 9 mg/day was given orally for 1 month, then reduce 3 mg every 3 months, with complete withdrawal after 1 year.	During this period, episodes of macrohematuria dramatically decreased, and UPCR and kidney function were maintained stable.	NA

UPCR, urinary protein-to-creatinine ratio; NA, not available.

### 5.2 The main findings reported in these studies

In 1986, corticosteroids were first reported for IgAN treatment, and budesonide was later used in 2011 [37,46]. The KDIGO guidelines suggest considering a 6-month course of glucocorticoid therapy for patients at high risk of progressive renal function loss with proteinuria exceeding 1 g/24 h, despite optimized supportive treatment for at least 90 days [12]. However, the use of glucocorticoids in IgAN remains a controversial topic within the medical community. A recent multicenter, double-blind RCT indicated that oral methylprednisolone, administered for a period of 6–9 months, significantly improved renal function or helped slow the progression to renal failure in patients suffering from IgAN [47]. The TESTING trial [17] found addition of steroid therapy to supportive treatment effectively reduced proteinuria and significantly increased remission rate compared to supportive treatment alone. In the comprehensive study, researchers found that the patients who used steroids alone experienced a significantly higher number of side effects. The STOP trial [16] showed despite significant decrease in urinary protein, there was no significant benefit in GFR decline with immunosuppressive therapy versus standard treatment.

It is critically important to recognize and address the potential for adverse events associated with oral steroid treatment, especially when administered at high doses. Targeted treatments that demonstrate a favorable risk-benefit ratio are urgently needed for patients suffering from IgAN who are at a high risk of progressing to end-stage renal disease. Smerud et al. reported the inaugural use of TRF-budesonide for the treatment of IgAN in an open-label, phase 2a trial [37]. This study included 16 patients suffering from IgAN who were administered TRF-budesonide, despite having received optimized RAS blockade or immunosuppressive/steroid therapy. Proteinuria was high (>500 mg/day, median: 1579 mg/day) before treatment. Patients received TRF-budesonide (8 mg/day) for 6 months, followed for 3 months. There was a median reduction in urine albumin of 529 mg/day during treatment (P = 0.04), peaking at 40 % after 2 months. Serum creatinine relative reduction was 6 % from baseline post-treatment (P = 0.003). Two individuals opted to discontinue their participation due to the onset of abdominal discomfort, with no glucocorticoid-related adverse effects observed. The study demonstrated significant reduction in proteinuria and good tolerability in IgAN patients treated with TRF-budesonide. Fellström et al., in an effort to gain a more comprehensive understanding of the safety and efficacy of TRF-budesonide in patients diagnosed with IgAN, embarked on a randomized, meticulously designed, double-blind, placebo-controlled phase 2b trial, aptly titled NEFIGAN [38]. This study included 149 patients suffering from IgAN. Patients received 16 mg/day or 8 mg/day of Nefecon or placebo for 9 months. The mean UPCR reduced by 27.3 % and 21.5 % in the 16 and 8 mg/day groups respectively (P = 0.0092 and P = 0.0290), while the placebo group increased by 2.7 %. 2 adverse events occurred (deep vein thrombosis and renal function deterioration) in the Nefecon treatment group. Subsequently, Ismail et al. [39] compared Budenofalk with corticosteroids in 18 IgAN patients for 24 months. Proteinuria reduced by 45 % in the TRF-budesonide group, compared to 11 % with corticosteroids (P = 0.009), showing budesonide significantly reduced proteinuria and hematuria in IgAN. Lafayette et al. designed the phase III NefIgArd trial [18] to further investigate TRF-budesonide's efficacy and safety in 182 IgAN patients. After 9 months' treatment, there was significant proteinuria reduction persisting 9 months post-drug discontinuation (−40.9 % from month 12–24 compared to placebo). The 2-year average eGFR showed statistically significant benefit of Nefecon over placebo (5.05 mL/min per 1.73 m^2^ (p < 0.0001). No serious adverse events occurred. In 2024, Zhang H et al. [41] conducted a two-year study on the efficacy and safety of Nefecon in patients with IgAN in mainland China, including 62 participants. The time-weighted average difference in eGFR over two years was 9.6 mL/min/1.73 m^2^, showing a significant advantage over the placebo group. Additionally, there was a reduction in UPCR by 31 % at 9 months and 43 % at 24 months. These results indicate that Nefecon has a substantial impact in the treatment of IgAN, with even greater therapeutic benefits observed in Chinese patients.

The United States Food and Drug Administration (FDA) approved TRF-budesonide (Nefecon/TarpeyoTM) on December 15, 2021. This marked a significant milestone as it was the first targeted drug approved specifically for adult primary IgAN. In Europe, the European Medicine Agency (EMA), a similar regulatory body responsible for evaluating and monitoring medicines, granted conditional marketing authorization for TRF-budesonide in 2022. With this authorization, TRF-budesonide can be marketed and sold in the European Union while additional data is collected to support its full authorization. As we await results of subsequent global phase 4 clinical trials, which are designed to evaluate the drug's efficacy and safety in real-world settings, these trials will provide more reliable evidence for the treatment of IgAN.

## 6 Application of budesonide in children with IgAN

In children with IgAN, the efficacy of immunosuppressive therapy remains a topic of debate due to concerns over the chronic progression of histological changes. Compared to adult nephrologists, pediatric nephrologists tend to prescribe steroid therapy more frequently, as well as ACEI/ARBs. However, the long-term use of steroid in children carries significant risks of toxicity, necessitating careful consideration and close monitoring throughout the treatment process. Use of TRF-budesonide in children hasn't been deeply studied, with few case reports published. Venettacci et al. [42] reported a successful case of pediatric IgAN treatment using TRF-budesonide in an 8-year-old boy. He had severe IgAN, nephrotic-range proteinuria, elevated serum creatinine, and high urine protein creatinine ratio (UPCR) despite systemic corticosteroids and ACEI/ARB. At 12 years old, systemic corticosteroids were discontinued, and he began 3 mg/day of TRF-budesonide increased to 6 mg/day after 4 weeks. After 6 months, there was a dramatic decrease in the UPCR, plummeting from a high of 520 mg/mmol to a much lower value of 69 mg/mmol Śladowska-Kozłowska et al. [44] described a case of a 13-year-old boy with IgAN experiencing renal failure, hypertension, and proteinuria. He was unresponsive to methylprednisolone, systemic corticosteroids, and ACEI/ARB, so was started on TRF-budesonide 15 mg/day along with mycophenolate mofetil (MMF) and ACEI/ARB. After 6 months, UPCR decreased below 100 mg/mmol, serum creatinine to 1.6 mg/dL. Budesonide was well-tolerated with no side effects. In 2023, Antonucci et al. [45] described a case of a 13-year-old boy with IgAN who had undergone multiple treatments, including steroids and RAAS inhibitor, but gross hematuria persisted and proteinuria worsened. He was then initiated on TRF-budesonide at 9 mg/day. One month later, gross hematuria resolved and proteinuria decreased. Due to cortisol suppression and difficulty in drug supply, TRF-budesonide was tapered by 3 mg every three months and discontinued after a year. During this time, gross hematuria frequency decreased and proteinuria and renal function remained stable. In three known pediatric case reports to date, TRF-budesonide has effectively reduced proteinuria and has a renoprotective effect, with a superior safety profile compared to systemic glucocorticoids. However, TRF-budesonide in children has not been subjected to large-scale, multicenter studies and lacks specific dosing or treatment duration. These limitations expose children to risk of side effects and limit TRF-budesonide use in pediatric patients. More RCTs, large-scale, and multicenter studies are needed to support safe expansion of TRF-budesonide for children with IgAN.

## 7 Comparison of budesonide with other alternative treatments

### 7.1 Comparison of budesonide with systemic corticosteroids

**Fig. 3:**
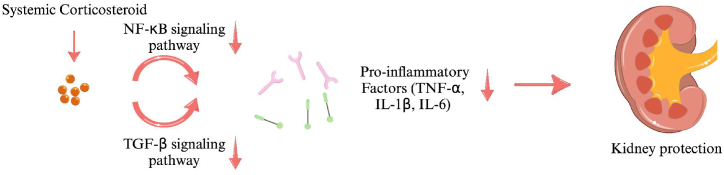
Potential mechanisms involved in the use of systemic corticosteroid in IgAN.

The current consensus is that glucocorticoids should be cautiously preserved and judiciously utilized solely for individuals diagnosed with IgAN who are identified as being particularly vulnerable to an accelerated progression towards end-stage renal disease (ESRD). The potential mechanisms of systemic corticosteroids use in IgAN are illustrated in Fig. 3. Budesonide is increasingly being recognized as a promising therapeutic option for IgAN, showing multiple positive outcomes compared to systemic corticosteroids with fewer negative effects. TRF-budesonide has a good safety profile, with most adverse events mild, non-serious, and reversible. In contrast, systemic corticosteroids can cause Cushingoid side effects, infections, and other serious adverse events. In 2024, Baragetti I et al. [48] conducted a multicenter cohort study on the safety of corticosteroid treatment for IgAN. Among 1,209 patients, a total of 119 adverse events (9.8 %) were reported. The study found that the incidence of serious adverse events during corticosteroid treatment, whether used alone or in combination with immunosuppressants, was lower in routine clinical practice compared to that observed in randomized clinical trials. However, the 2021 KDIGO guidelines [12] do not endorse budesonide due to lack of reporting and few studies requiring larger-scale randomized trials. In the latest 2024 KDIGO draft, Nefecon is recommended for patients with IgAN who are at risk of progressive loss of renal function. It remains to be seen, in light of available evidence, if this treatment is indeed safer and more effective than systemic glucocorticoids, the standard of care in many cases. Additionally, budesonide may be more costly than other treatments. The price could impact market entry for all medications, including TRF-budesonide.

### 7.2 Comparison of budesonide with immunosuppressants

According to the latest 2024 KDIGO draft, azathioprine is not recommended for use in IgAN due to a lack of supporting evidence for its efficacy. Cyclophosphamide is also not recommended for use in IgAN, except rapidly progressive IgAN. MMF and hydroxychloroquine are recommended for Chinese patients with IgAN. Compared to TRF-budesonide, immunosuppressants—excluding azathioprine and cyclophosphamide—are primarily suited for patients exhibiting crescentic lesions and those with rapidly progressive clinical trajectories. The efficacy and safety of MMF and hydroxychloroquine (HCQ) warrant further investigation for substantiation. A RCT involving 40 Chinese patients with IgAN over a follow-up period of up to six years suggested that MMF treatment may yield a transient and partial reduction in proteinuria in the short term, while potentially offering renal protection in the long term [49]. Conversely, a three-year prospective RCT conducted within European and American cohorts revealed that a regimen of MMF at 2 g/d for three years failed to demonstrate beneficial effects on renal function, outcomes, or improvements in proteinuria among IgAN patients predisposed to progressive disease [50]. Consequently, in light of the conflicting findings across diverse ethnic groups and the generally modest sample sizes of existing published RCTs, the 2021 KDIGO guidelines recommend MMF solely as an alternative treatment to glucocorticoids for Chinese patients. A double-blind, randomized, placebo-controlled phase II clinical study [51] encompassing 60 subjects yielded noteworthy results, highlighting a significant difference in the percentage change of proteinuria between the HCQ and placebo groups at six months (−48.4 % vs. 10.0 %, P < 0.001). The median proteinuria level in the HCQ group was markedly lower than that of the placebo group (0.9 g/d vs. 1.9 g/d, P = 0.002), underscoring HCQ's notable efficacy in reducing proteinuria and its safety in treating IgAN. However, the study faced limitations due to a small sample size, brief treatment duration, and the absence of post-treatment observations. Thus, the safety and efficacy of HCQ in the management of IgAN necessitate validation through larger-scale, long-term follow-up studies with ethnically diverse populations.

In summary, both TRF-budesonide and immunosuppressants possess distinct advantages and disadvantages in the treatment of IgAN, yet caution is imperative regarding their potential side effects. More researches are needed to compare the efficacy and safety between budesonide and immunosuppressive therapies. Future research should delve deeper into the synergistic application of these two therapeutic modalities to formulate a more holistic treatment strategy for patients afflicted with IgAN.

### 7.3 Comparison of budesonide with Telitacicept

Telitacicept is an innovative recombinant fusion protein composed of a transmembrane activator, a calcium modulator, and a ligand interaction domain derived from cyclophilin, in addition to the Fc portion of IgG. By interacting with Blys and APRIL—two critical factors influencing B cell survival and function—Telitacicept effectively curtails the development, maturation, and secretion of antibodies by B cells. This mechanism subsequently reduces the levels of Gd-IgA1 and anti-Gd-IgA1 autoantibodies, targeting the treatment of IgAN at its source.

The results of a Phase II clinical study evaluating the efficacy and safety of Telitacicept in the treatment of IgAN [52] demonstrated that Telitacicept effectively reduced proteinuria in high-risk IgAN patients. Additionally, no serious adverse events were reported, highlighting its commendable safety profile; however, further large-scale studies are essential to validate its efficacy and safety in the future. TRF-budesonide is an oral medication, whereas Telitacicept necessitates injection for administration, rendering TRF-budesonide more convenient by comparison. Both have demonstrated potential in treating IgAN and are anticipated to emerge as new strategies for its management in the future.

## 8 Long-term efficacy and safety of budesonide

Based on evidence, IgAN patients may benefit significantly from TRF-budesonide treatment, which targets the disease core by suppressing Gd-IgA1-IgG production. It reduces proteinuria and stabilizes renal function, delaying disease progression. Budesonide may cause hypertension, muscle cramps, acne, weight gain, dermatitis, indigestion, dyspnea, fatigue, and excessive hair growth. Adverse events are usually mild and reversible.

A recent study [53] evaluated the adverse reactions of TRF-budesonide, including a total of 1,515 IgAN patients. Through disproportionality analysis (DPA), 5 System Organ Classes (SOCs) and 23 Preferred Terms (PTs) were identified as positive signals at the SOC and PT levels. Among these signals, 4 PTs (fatigue, malaise, product dose omission issues, and anxiety) were newly identified adverse events in this study. No adverse events related to death were found among all identified PTs, and no PTs were classified as high clinical priority. Acne, hypertension, facial swelling, and weight gain were considered moderate clinical priority events. These findings demonstrate the good safety profile of TRF-budesonide, providing positive safety evidence for its use in the treatment of IgAN.

Given this evidence, different patients may variably respond to budesonide, with some responding well to treatment while others require dose adjustments or alternative therapeutic strategies. During long-term treatment, regular monitoring of renal function, blood pressure, proteinuria levels, and potential side effects is necessary, with treatment plan adjustments as needed. Currently, budesonide use in IgAN is under investigation, and future clinical trial results may provide clearer information on long-term efficacy and safety.

The possibility of relapse after withdrawing TRF-budesonide in the treatment of IgAN is still not fully understood due to limited research and clinical experience. The likelihood of relapse is influenced by several factors, including the underlying disease activity of the patient, the duration and effectiveness of the treatment, and individual patient differences. To more accurately assess the risk of relapse after withdrawing TRF-budesonide, further long-term follow-up studies and clinical trial data are needed in the future.

## 9 Future prospects of budesonide in IgAN

Although the potential of TRF-budesonide in the treatment of IgAN has been demonstrated, several issues require further investigation. Firstly, its long-term efficacy and safety need to be explored. Current studies primarily focus on short-term effects, but the impact of TRF-budesonide on long-term renal outcomes remains under-examined. This includes the durability of its renal protective effects, potential side effects from prolonged use, and the possibility of relapse after discontinuation. Secondly, the strategy of combining TRF-budesonide with other medications warrants further study. Whether it can be effectively used in conjunction with other therapies, such as MMF or ACEI/ARBs, to achieve superior therapeutic outcomes, still needs to be validated through additional randomized controlled trials. Finally, assessing the efficacy of TRF-budesonide across different racial groups is important. For instance, whether there are differences in efficacy and safety between Chinese patients and those from Western populations is a key area for future research.

In summary, future directions in the treatment of IgAN should focus on developing personalized treatment plans, exploring multi-target combination therapy strategies, and evaluating the long-term safety and cost-effectiveness of medications. These efforts aim to improve prognosis and enhance the quality of life for patients with IgAN.

## 10 Conclusion

In the current medical landscape, there is a consensus among experts that ACEIs and ARBs represent the gold standard for first-line treatment options for IgAN. This recommendation is supported by a substantial body of research demonstrating that these medications are effective in managing the condition.

In recent years, TRF-budesonide has garnered increasing attention as a novel treatment option for IgAN. This medication works by specifically targeting the GALT to reduce the production of Gd-IgA1, thereby intervening in the core pathological mechanism of IgAN. This approach is distinctly different from traditional treatment methods. A growing body of research indicates that TRF-budesonide not only significantly reduces proteinuria levels but also provides sustained protection of kidney function. Furthermore, studies have demonstrated that TRF-budesonide exhibits a favorable safety profile, markedly reducing systemic side effects associated with glucocorticoid exposure, such as infection risk, metabolic disturbances, and osteoporosis, when compared to systemic corticosteroids.

Nevertheless, the widespread application of TRF-budesonide in the treatment of IgAN still faces several challenges. Firstly, the number of patients receiving TRF-budesonide therapy in clinical settings is limited. Secondly, there is a lack of high-quality data regarding the long-term safety and efficacy of TRF-budesonide. As such, despite its demonstrated efficacy in short- and medium-term studies, its long-term outcomes, such as ESRD, require further validation.

To conclude, while ACEIs and ARBs remain the cornerstone of IgAN treatment, TRF-budesonide, as a more targeted and innovative medication, shows potential to become an additional first-line treatment option. Given the current research advancements, there is an urgent need for more rigorously designed randomized controlled trials to comprehensively evaluate TRF-budesonide. Through further evidence-based medical research and the accumulation of long-term follow-up data, TRF-budesonide is expected to play a more significant role in the future management of IgAN, offering patients more personalized and precise treatment options.

## CRediT authorship contribution statement

**Feifan Qi:** Writing – review & editing, Writing – original draft, Methodology, Data curation, Conceptualization. **Hui-qin Zeng:** Supervision, Investigation, Data curation. **Jian-jiang Zhang:** Writing – review & editing, Supervision, Methodology, Conceptualization.

## Availability of data and materials

No new data were generated or analysed in support of this research.

## Ethics approval and consent to participate

Not applicable.

## Fundings

This work was supported by the Medical Science and Technology Research Project of Henan Province (No. SBGJ202302078).

## Declaration of competing interest

The authors declare the following financial interests/personal relationships which may be considered as potential competing interests:Jianjiang Zhang reports financial support was provided by Medical Science and Technology Research Project of Henan Province. If there are other authors, they declare that they have no known competing financial interests or personal relationships that could have appeared to influence the work reported in this paper.
